# Characterization of the complete chloroplast genome of *Liriope platyphylla* (Asparagaceae: Nolinoideae) isolated in Korea

**DOI:** 10.1080/23802359.2020.1787898

**Published:** 2020-07-20

**Authors:** Ji-Hun Jang, Ho-Kyung Jung, Ho Park, Kyung-Min Lee, Ji-Kyung Kim, Tae-Yeon Hwang, Hyun-Woo Cho

**Affiliations:** National Development Institute of Korean Medicine, Jangheung-gun, South Korea

**Keywords:** Chloroplast genome, *Liriope platyphylla*, phylogenetic tree analysis

## Abstract

*Liriope platyphylla* is used as an important medicinal plant for fatigue, cough, and inflammation in South Korea. Here, we report the complete chloroplast genome of *L. platyphylla*. The total genome size of the chloroplast is 157,076 bp with a large single-copy region (LSC: 85,374 bp), a small single-copy region (SSC: 18,748 bp), and inverted repeat regions (IRa and IRb: 26,477 bp). The GC content of the *L. platyphylla* chloroplast was 37.6%. The cp genome encoded a set of 129 genes, including 83 protein-coding genes, 38 tRNA genes, and 8 rRNA genes. The phylogenetic tree analysis indicated that *L. platyphylla* is closely related to *L. spicata*.

*Liriope platyphylla* is an herbaceous plant belonging to the Asparagaceae family and is widely distributed in East Asia (Korea, China, Japan, and Taiwan) (Shin 2002). *Liriope platyphylla* is common in the southern region of South Korea and has been used in traditional medicine for nutritional tonics, expectorants, and diuresis (Han 1993). Recent studies of *L. platyphylla* have reported anti-inflammatory action (Kim et al. 2016), immunomodulatory effects (Kim 2003), protective effects of the liver (Park et al. 2019) and lungs (Lee et al. 2011), anti-cancer effects (Wang et al. 2013), and memory enhancement effects (Kang and Lee 2006).

In this study, we revealed the complete chloroplast sequence of *L. platyphylla* and phylogenetic information of the species. Fresh leaves (*L. platyphylla*) were collected from Anyang-myeon, Jangheung-gun, Korea (34°40′1″N, 126°56′42″E). A voucher specimen (TKMII-32) was deposited at the Medicinal Crops Seed Supply Center of the National Institute for Korean Medicine Development (NIKOM). The whole chloroplast DNA was isolated using the DNeasy Plant mini kit (QIAGEN, Hilden, Germany), and the raw read sequence (10,629,338 bp) was achieved by Illumina platform (HiSeq 2500 & NovaSeq) at Genotech Inc. (Yuseong-gu, Daejeon, Korea). Raw reads were assembled via NOVOPlasty v2.6.7 (Dierckxsens et al. 2017). The assembled genome was annotated using the Dual Organellar Genome Annotator (Dogma; Wyman et al. 2004) and the Chloroplast Genome Annotation, Visualization, Analysis, and GenBank Submission Tool (CPGAVAS2; Shi et al. 2019).

The complete chloroplast genome of *L. platyphylla* was 157,076 bp in length and consisted of two single-copy regions (large single-copy region (LSC) with 85,374 bp, and small single-copy region (SSC) with 18,748 bp) and a pair of inverted repeat (IR) regions (IRa and IRb with 26,447 bp). The cp genome encoded a total of 129 genes, including 83 protein-coding genes, 38 tRNA genes, and 8 rRNA genes. The total GC content of the chloroplast genome was 37.6%, with corresponding contents in the LSC, SSC, and IR regions of 35.6, 31.2, and 43.0%, respectively. The alignment was conducted using MAFFT (Katoh and Standley 2013). The phylogenetic tree (neighbor joining tree) was constructed using CLC Main Workbench 7 with a bootstrap set to 10,000 (Figure 1). The phylogenetic tree indicated that *L. platyphylla* was most similar to *L. spicata.*

**Figure 1.**
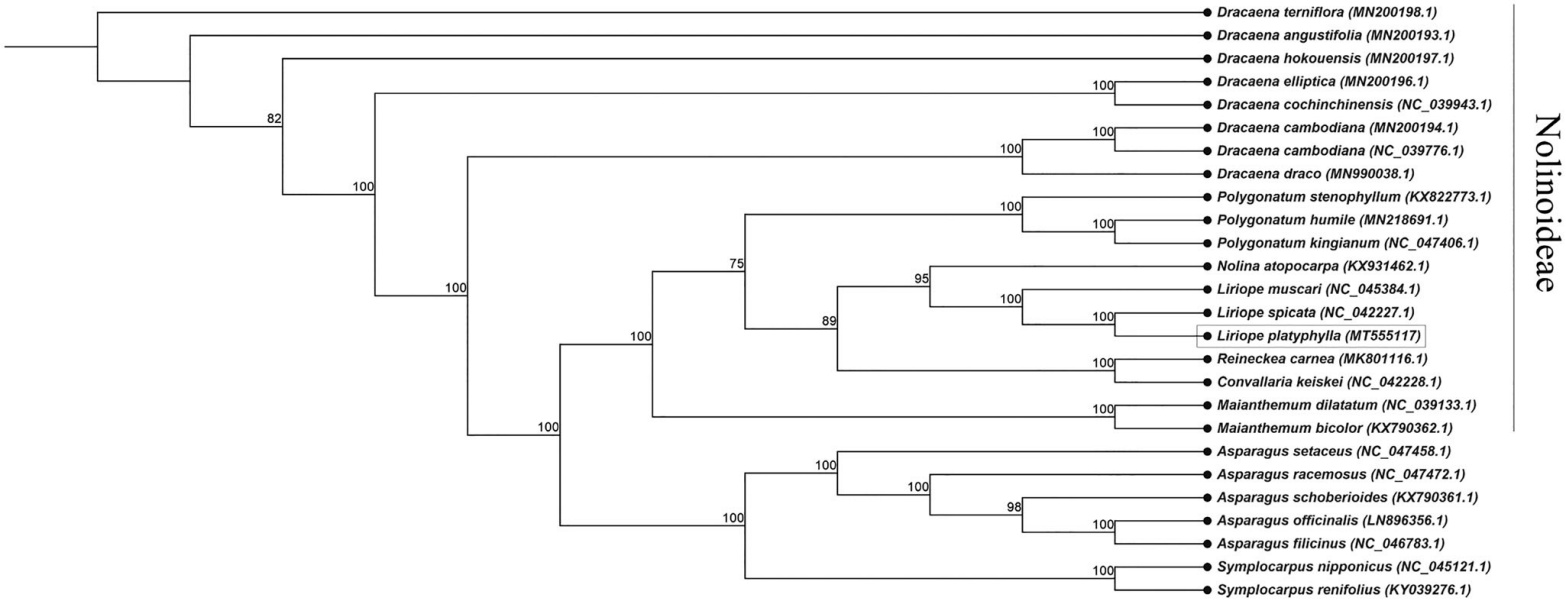
The neighbor joining tree (bootstrap repeat = 10,000) inferred from 26 plastid genomes.

## Data Availability

The data of this study are publicly published in NCBI's GenBank (https://www.ncbi.nlm.nih.gov/genbank/), reference number MT555117.
